# PARP1 expression in soft tissue sarcomas is a poor‐prognosis factor and a new potential therapeutic target

**DOI:** 10.1002/1878-0261.12522

**Published:** 2019-06-07

**Authors:** François Bertucci, Pascal Finetti, Audrey Monneur, Delphine Perrot, Christine Chevreau, Axel Le Cesne, Jean‐Yves Blay, Olivier Mir, Daniel Birnbaum

**Affiliations:** ^1^ Predictive Oncology Laboratory, Marseille Cancer Research Center (CRCM), Institut Paoli‐Calmettes, U1068 INSERM, U7258 CNRS Aix‐Marseille University Marseille France; ^2^ Department of Medical Oncology Institut Paoli‐Calmettes Marseille France; ^3^ French Sarcoma Group Lyon France; ^4^ Department of Medical Oncology, IUCT‐Oncopole Institut Claudius‐Regaud Toulouse France; ^5^ Department of Medical Oncology Gustave Roussy Villejuif France; ^6^ Department of Medical Oncology Centre Léon Bérard Lyon France; ^7^ Department of Ambulatory Care Gustave Roussy Villejuif France

**Keywords:** PARP inhibitor, PARP1 expression, prognosis, soft tissue sarcoma, survival

## Abstract

Soft tissue sarcomas (STSs) are aggressive tumors with few efficient systemic therapies. Poly(ADP‐ribose) polymerase‐1 (PARP1) inhibitors represent an emerging therapeutic option in tumors with genomic instability. The genomics of STSs is complex in more than half of cases, suggesting a high level of inherent DNA damage and genomic instability. Thus, STSs could be efficiently targeted with PARP inhibitors. Promising preclinical results have been reported, but few data are available regarding PARP1 expression in clinical samples. We examined *PARP1* mRNA expression in 1464 clinical samples of STS, including 1432 primary tumors and 32 relapses, and searched for correlations with clinicopathological features, including metastasis‐free survival (MFS). Expression was heterogeneous across the samples, not different between primary and secondary tumors, and was correlated to *PARP1* DNA copy number. In the 1432 primary tumors, the ‘PARP1‐high’ samples were associated with younger patients, more frequent locations at the extremities, superficial trunk and head and neck, more leiomyosarcomas and other STSs and less liposarcomas and myxofibrosarcomas, more grade 3, more high‐risk CINSARC tumors, and more ‘chromosomically instable’ tumors. They were associated with shorter MFS, independently of other significant prognostic features, including the CINSARC signature. We found a strong involvement of genes overexpressed in the ‘PARP1‐high’ samples in cell cycle, DNA replication, and DNA repair. *PARP1* expression refines the prediction of MFS in STSs, and similar expression exists in secondary and primary tumors, supporting the development of PARP1 inhibitors.

**Abbreviations**
CNA: copy number alteration
FNCLCC: Fédération Nationale des Centres de Lutte Contre le Cancer
GO: Gene ontology
LR: likelihood ratio
MFS: metastasis‐free survival
MPNSTs: malignant peripheral nerve sheath tumors
PARP1: Poly(ADP‐ribose) polymerase‐1
SSBs: single‐strand breaks
STSs: soft tissue sarcomas
TCGA: The Cancer Genome Atlas

## 1 Introduction

Soft tissue sarcomas (STSs) are rare, severe, and heterogeneous tumors including many different pathological subtypes (Casali *et al.*, 2018). Surgery is the main treatment in early stages, but more than 40% of operated patients will ultimately experience metastatic relapse and die. The survival benefit of adjuvant anthracycline‐based chemotherapy remains unproven, likely in part because of the absence of accurate prognostic features and predictors of response to chemotherapy. Identification of new prognostic features such as the promising CINSARC gene expression signature (Chibon *et al.*, 2010) is warranted. In patients with metastatic disease not amenable to curative‐intent surgery, the first‐line systemic treatment involves palliative chemotherapy that has very little change over the three past decades and remains based on doxorubicin. After intolerance or failure, the second‐line therapies include chemotherapies (ifosfamide, dacarbazine, trabectedin, eribulin) and targeted therapy (pazopanib), but the results remain disappointing. Clearly, the improvement of systemic therapies and identification of new prognostic and therapeutic targets are crucial.

An emerging therapeutic option in oncology concerns PARP inhibitors (Lim and Tan, 2017). Poly(ADP‐ribose) polymerase‐1 (PARP1) is a nuclear chromatin‐associated protein involved in several biological processes including cell proliferation, apoptosis, malignant transformation, transcriptional regulation, and DNA repair. It is essential to the base excision repair of DNA single‐strand breaks (SSBs). In response to DNA damage, PARP1 senses and binds to DNA nicks and breaks, resulting in activation of catalytic activity, causing poly(ADP)ribosylation of PARP1 itself, as well as other acceptor proteins such as histones and topoisomerases. This modification stimulates the recruitment and activity of other components of DNA repair pathways (Ame *et al.*, 2004). In its absence, DNA SSBs accumulate and degenerate to DNA double‐strand breaks, which are not appropriately repaired if the BRCA pathway is deficient or dysfunctional. This is thought to explain the exquisite sensitivity to PARP inhibitors of tumors with BRCA inactivation, a concept called ‘synthetic lethality’ (Bryant *et al.*, 2005; Farmer *et al.*, 2005). Today, different PARP inhibitors are marketed or in development in advanced solid tumors (Lim and Tan, 2017) with BRCAness features.

Although STSs do not have a characterized defect in BRCA1/2, their genomics is complex in more than half of the cases, suggesting genomic instability and eventual possible deficiency in DNA damage repair and a high level of inherent DNA damage, as recently reported for leiomyosarcomas (Chudasama *et al.*, 2018). Thus, STSs could be efficiently targeted with PARP inhibitors to drive cells to synthetic lethality. In sarcomas, promising preclinical data have been reported, notably in Ewing sarcoma and in STSs (Chudasama *et al.*, 2018; Laroche *et al.*, 2017; Stewart *et al.*, 2014; Vormoor and Curtin, 2014). Recently, the safety of the combination of trabectedin chemotherapy and olaparib PARP inhibitor in second‐line or further‐line has been shown in patients with advanced sarcomas (Grignani *et al.*, 2018), with a promising 18% partial response in patients with STS. The response rate and progression‐free survival were higher in patients with high *PARP1* tumor expression, confirming their preclinical findings (Pignochino *et al.*, 2017).

However, very few data are available in the literature regarding *PARP1* expression in clinical STS samples. To our knowledge, only two studies are available and concern only 91 malignant peripheral nerve sheath tumors (MPNSTs) (Kivlin *et al.*, 2016) and 112 STSs (Kim *et al.*, 2016). To fill this gap, we examined *PARP1* mRNA expression in a series of 1464 clinical samples of STS, including 1432 primary tumors and 32 relapses, and searched for correlations with clinicopathological features, including metastasis‐free survival (MFS).

## 2 Materials and methods

### 2.1 Soft tissue sarcoma samples and data sets

We retrospectively gathered clinicopathological and gene expression data of clinical STS samples from 16 public data sets (Baird *et al.*, 2005; Barretina *et al.*, 2010; Beck *et al.*, 2010; Cancer Genome Atlas Research Network, 2017; Chibon *et al.*, 2010; Detwiller *et al.*, 2005; Gibault *et al.*, 2011; Gobble *et al.*, 2011; Hajdu *et al.*, 2010; Henderson *et al.*, 2005; Nakayama *et al.*, 2007; Nielsen *et al.*, 2002; Renner *et al.*, 2013; Skubitz *et al.*, 2012; West *et al.*, 2005; Ylipaa *et al.*, 2011). Sets and raw data were collected from the National Center for Biotechnology Information (NCBI)/GenBank GEO and ArrayExpress databases and authors’ web sites (Table S1). The selection of data sets was based according to the availability of clinical and expression data, including *PARP1* expression measurement. Samples had been profiled using DNA microarrays or RNASeq. The pooled data set contained a total of 1464 clinical samples of primary STS, including 1432 primary STSs and 32 STS relapses. These relapse samples were included in order to compare the PARP1 mRNA expression level between primary tumors and relapse samples, since these later will be the first candidates to PARP inhibitors in their clinical development. We also collected *PARP1* DNA copy number, DNA methylation, and DNA mutational data of 224 STS primary tumors profiled in the The Cancer Genome Atlas (TCGA) data set (Cancer Genome Atlas Research Network, 2017) using SNP array and whole‐exome sequencing, respectively.

### 2.2 Gene expression data analysis

The pre‐analytic processing first included normalization of each data set separately, by using robust multichip average (Irizarry *et al.*, 2003) with the nonparametric quantile algorithm for the raw Affymetrix data and quantile normalization for the available processed non‐Affymetrix microarray data. Normalization was done in R using Bioconductor and associated packages. Then, we mapped hybridization probes across the different technological platforms as reported (Bertucci *et al.*, 2014). When multiple probes mapped to the same GeneID, we retained the one with the highest variance in each data set. We log_2_‐transformed the already normalized TCGA RNAseq data. Next, the batch effects were corrected across the 16 studies using z‐score normalization. Briefly, for each expression value in each study separately, all values were transformed by subtracting the mean of the gene in that data set divided by its standard deviation, mean and standard deviation (SD) being measured on leiomyosarcoma samples. We applied to each data set separately two gene expression signatures: the CINSARC signature (Chibon *et al.*, 2010) and the Carter’s chromosomal instability signature (Carter *et al.*, 2006).

To decipher the biological pathways associated with *PARP1* expression in STSs, we applied a supervised analysis to expression profiles of the 224 TCGA samples (learning set) to search for genes differentially expressed between the ‘PARP1‐high’ vs ‘PARP1‐low’ classes (cut‐off defined as the median expression level across all samples). We used a moderated t‐test with empirical Bayes statistic included in the limma R packages. False discovery rate (Hochberg and Benjamini, 1990) was applied to correct the multiple testing hypothesis: The significant genes were defined by *p* < 1%, *q* < 1%, and fold change superior to |1.5×|. The robustness of the resulting gene list was tested in the validation set of 1208 remaining samples (592 ‘PARP1‐low’ samples and 616 ‘PARP1‐high’ samples) by computing for each tumor a metagene‐based prediction score defined by the difference between the ‘metagene PARP1‐up’ (mean expression of all genes upregulated in the ‘PARP1‐high’ class) and the ‘metagene PARP1‐low’ (mean expression of all genes upregulated in the ‘PARP1‐low’ class). This score was then compared between the ‘PARP1‐high’ and ‘PARP1‐low’ samples. Ontology analysis of the resulting gene list was based on Gene ontology (GO) biological processes of the Database for Annotation, Visualization and Integrated Discovery (DAVID; http://david.abcc.ncifcrf.gov/).

### 2.3 Statistical analysis

Correlations between the *PARP1* expression‐based classes (low vs high) and the clinicopathological factors were calculated with Student’s *t*‐test for the continuous variables and the Fisher’s exact test for the binary variables. Our primary endpoint, MFS, was calculated from the date of diagnosis until the date of metastatic relapse. The follow‐up was measured from the date of diagnosis to the date of last news for event‐free patients. Survival was calculated using the Kaplan–Meier method, and curves were compared with the log‐rank test. Univariate and multivariate analyses were done using Cox regression analysis (Wald test). The variables tested in univariate analysis included the PARP1‐based classification (low vs high), patients’ age and gender, pathological type, grade, and tumor size, depth, tumor site, and the CINSARC risk (high vs low). Multivariate analysis incorporated all variables with a *P*‐value inferior to 5% in univariate analysis. The likelihood ratio (LR) tests were used to assess the prognostic information provided beyond that of the CINSARC signature, assuming a chi‐square distribution. Changes in the LR values (LR‐∆*X* ^2^) measured quantitatively the relative amount of information of one model compared with another. All statistical tests were two‐sided at the 5% level of significance. Statistical analysis was done using the survival package (version 2.30) in the r software (version 2.15.2) (R Foundation for Statistical Computing, Vienna, Austria). The paper was written in accordance with reporting recommendations for tumor marker prognostic studies (REMARK) criteria (McShane *et al.*, 2005).

## 3 Results

### 3.1 Patients’ characteristics

Gene expression profiles of 1432 clinical samples of STS primary tumors including *PARP1* expression level were available. Their characteristics are summarized in Table 1. The median patients’ age was 63 (range, 2–93) years. The sex ratio was balanced, with 49% of females. The most frequent anatomical sites were extremities, followed by internal trunk; 84% of tumors were deeply seated, below or through the superficial fascia. As expected, the most frequent pathological types were liposarcomas, leiomyosarcomas, and undifferentiated sarcomas. The median pathological tumor size on the operative specimen was 9 cm. Most of the samples were Fédération Nationale des Centres de Lutte Contre le Cancer (FNCLCC) grade 3, and 47% of samples were classified as high risk according to the CINSARC signature and 56% as chromosomically instable according to the Carter’s signature.

**Table 1:** Clinicopathological characteristics and correlations with the PARP1‐based classification.. FNCLCC, Fédération Nationale des Centres de Lutte Contre le Cancer

Characteristics	All	PARP1 classes		*P*‐value
		PARP1‐low	PARP1‐high	
Age, median				
Years (range)	63	65 (16.16–93)	60 (2–91)	**2.18E‐04**
Gender				
Female	331 (49%)	157 (47%)	174 (51%)	0.249
Male	346 (51%)	180 (53%)	166 (49%)	
Tumor site				
Extremity	207 (42%)	98 (40%)	109 (44%)	**1**.**90E‐02**
Internal trunk	195 (39%)	112 (45%)	83 (33%)	
Superficial trunk	84 (17%)	35 (14%)	49 (20%)	
Head and neck	9 (2%)	2 (1%)	7 (3%)	
Depth				
Deep	195 (84%)	115 (84%)	80 (84%)	1
Superficial	37 (16%)	22 (16%)	15 (16%)	
Pathological type				
Leiomyosarcoma	329 (23%)	142 (20%)	187 (27%)	**2**.**39E‐11**
Liposarcoma	472 (34%)	287 (40%)	185 (27%)	
Undifferentiated sarcoma	326 (23%)	166 (23%)	160 (23%)	
Myxofibrosarcoma	105 (7%)	64 (9%)	41 (6%)	
Other	174 (12%)	56 (8%)	118 (17%)	
Pathological tumor size, median				
cm (range)	9 (1.2–39.5)	10 (1.2–39.5)	8 (1.6–30)	0.093
Pathological FNCLCC grade				
1–2	163 (41%)	102 (48%)	61 (32%)	**1**.**57E‐03**
3	238 (59%)	110 (52%)	128 (68%)	
CINSARC risk				
Low	752 (53%)	421 (58%)	331 (47%)	**3**.**62E‐05**
High	680 (47%)	306 (42%)	374 (53%)	
Carter's signature				
Chromosomal stability	645 (44%)	419 (57%)	226 (31%)	**4**.**56E‐24**
Chromosomal instability	818 (56%)	314 (43%)	504 (69%)	
Metastatic events				
Number of patients (%)	209 (31%)	97 (26%)	112 (37%)	**1**.**99E‐03**
5‐year MFS				
% (95% CI)	63% (59–68)	69% (64–75)	56% (49–63)	**5**.**84E‐04**

*P*‐values in bold mean significant *P*‐values.

### 3.2 *PARP1* expression in soft tissue sarcomas

**Figure 1:**
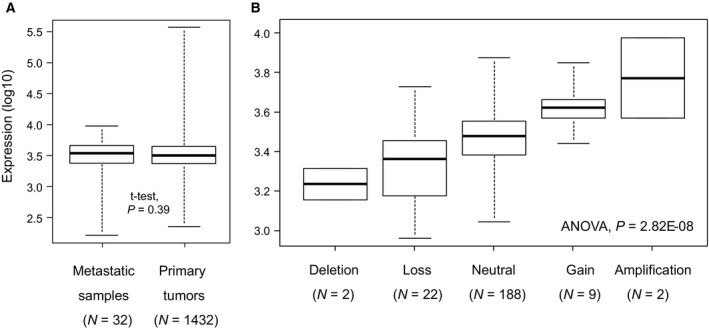
*PARP1* expression in STS. (A) Box plots showing *PARP1* mRNA expression level (log_10_) in 1432 STS primary tumors and 32 STS metastases. For each box plot, median and ranges are indicated. (B) Similar to (A) but applied to the 224 TCGA STS primary tumors and according to *PARP1* DNA copy number (SNP array).

*PARP1* mRNA expression was heterogeneous through the 1432 primary tumors with a three‐decade range of values, and it was similar between primary and secondary tumors (Fig. 1A). We searched for correlation between *PARP1* expression and DNA alterations at different levels (copy number, methylation, and mutation of *PARP1* gene on chromosome 1) that were simultaneously annotated in the 224 TCGA samples. No sample showed *PARP1* methylation or mutation. By contrast, 35 samples (16%) showed copy number alteration (CNA), including two deletions (homozygous losses: log_2_ ratio>|0.5|), 22 heterozygous losses, nine gains, and two amplifications (log_2_ ratio>|1|). There was a positive correlation between *PARP1* mRNA expression and DNA copy number, with increasing expression from samples with deletion, then loss, then no CNA, then gain, and finally amplification (*P = *2.82E‐08, ANOVA; Fig. 1B).

### 3.3 *PARP1* expression and clinicopathological characteristics

Within the 1432 primary tumors, and when compared with the ‘PARP1‐low’ class, the ‘PARP1‐high’ class was associated (Table 1) with younger patients’ age (*P* = 2.18E‐04, Student’s *t*‐test) and with (Fisher’s exact test) more frequent tumor locations at the extremities, superficial trunk and head and neck and less internal trunk locations (*P* = 1.90E‐02), pathological subtypes with more leiomyosarcomas and other STSs and less liposarcomas and myxofibrosarcomas (*P* = 2.39E‐11), higher pathological grade 3 (*P* = 1.57E‐03), high‐risk CINSARC class (*P* = 3.62E‐05), and higher Carter’s signature‐based chromosomal instability (*P* = 4.56E‐24). There was no correlation with patients’ gender, depth location, and pathological tumor size.

### 3.4 *PARP1* expression and metastasis‐free survival

**Figure 2:**
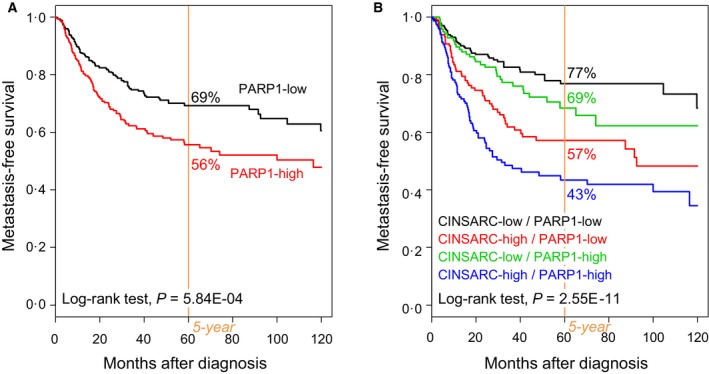
MFS in patients with STS according to *PARP1* expression. (A) Kaplan–Meier MFS curves in all patients with STS, according to the PARP1‐based classification (‘PARP1‐low’ and ‘PARP1‐high’ classes). (B) Similar to A, but according to the 4‐class classification based on both *PARP1* expression and CINSARC signature.

Metastasis‐free survival data were available for 678 nonmetastatic operated patients. The median follow‐up was 32 months (range, 1–222); 209 patients displayed a metastatic relapse, and the 5‐year MFS was 63% (95% CI: 59–68). The clinical outcome was different between the two PARP1‐based classes, with 112 events (37%) in the ‘PARP1‐high’ class (*N* = 303) vs 97 events (26%) in the ‘PARP1‐low’ class (*N* = 375; *P* = 1.99E‐03, Fisher’s exact test; Table 1). The 5‐year MFS was 56% (95% CI: 49–63) vs 69% (95% CI: 64–75), respectively (*P* = 5.84E‐04; Fig. 2A).

In univariate analysis for MFS (Table 2), the hazard ratio (HR) for metastatic relapse was 1.60 (95% CI: 1.22–2.11) in the ‘PARP1‐high’ class when compared to the ‘PARP1‐low’ class (*P* = 6.56E‐04, Wald test). Other variables associated with MFS included the pathological type (*P* = 1.35E‐06) and the CINSARC classification (*P* = 2.03E‐10). In multivariate analysis (Table 2), *PARP1* expression remained significant (*P* = 1.86E‐02, Wald test), as well as pathological type, and CINSARC classification, suggesting independent prognostic value. Indeed, PARP1 expression affected the clinical outcome of the CINSARC classes (Fig. 2B): The 5‐year MFS was 77% (95% CI: 70–84) in the ‘CINSARC‐low’/‘PARP1‐low’ class, 69% (95% CI: 60–79) in the ‘CINSARC‐low’/‘PARP1‐high’, 57% (95% CI: 48–67) in the ‘CINSARC‐high’/‘PARP1‐low’ class, and 43% (95% CI: 35–54) in the ‘CINSARC‐high’/‘PARP1‐high’ class (*P* = 2.55E‐11, log‐rank test). Such prognostic complementarity was confirmed using the LR tests: *PARP1* expression added information to that provided by CINSARC (LR‐∆*X* ^2^=4.7, *P* = 3.08E‐02). Similarly, *PARP1* expression affected the MFS in two out of three major pathological types of STS (Fig. S1): liposarcomas with 71% 5‐year MFS in the ‘PARP1‐low’ class vs 57% in the ‘PARP1‐high’ class (*P* = 3.17E‐02, log‐rank test) and undifferentiated sarcomas with respective 5‐year MFs equal to 80% vs 59% (*P* = 7.76E‐03, log‐rank test). The difference was not significant in leiomyosarcomas. Of note, the same prognostic analysis using *PARP1* expression as continuous variable showed the same independent prognostic value (Table 2).

**Table 2:** Univariate and multivariate prognostic analyses for MFS. FNCLCC, Fédération Nationale des Centres de Lutte Contre le Cancer.

Characteristics	Univariate			Multivariate			Multivariate		
	*n*	HR (95% CI)	*P*‐value	*n*	HR (95% CI)	*P*‐value	*n*	HR (95% CI)	*P*‐value
Age									
Years	371	1.00 (0.99–1.01)	0.902						
Gender									
Male vs Female	371	1.02 (0.70–1.49)	0.909						
Tumor site									
Head and neck vs Extremity	382	0.00 (0.00 – Inf)	0.66						
Internal trunk vs Extremity		0.77 (0.50–1.18)							
Superficial trunk vs Extremity		0.81 (0.47–1.40)							
Depth									
Superficial vs Deep	196	0.78 (0.38–1.61)	0.495						
Pathological type									
LipoS. vs LeiomyoS.	678	0.48 (0.35–0.67)	**1**.**35E**–**06**	678	0.65 (0.46–0.93)	**1**.**67E**–**02**	678	0.63 (0.45–0.89)	**9**.**36E**–**03**
MyxofibroS. vs LeiomyoS.		0.45 (0.24–0.86)		678	0.53 (0.28–1.01)	**0**.**052**	678	0.52 (0.27–0.98)	**4**.**26E**–**02**
Undifferentiated S. vs LeiomyoS.		0.43 (0.30–0.61)		678	0.49 (0.34–0.70)	**9**.**87E**–**05**	678	0.47 (0.33–0.68)	**4**.**64E**–**05**
Other vs LeiomyoS.		0.21 (0.08–0.57)		678	0.27 (0.10–0.74)	**1**.**13E**–**02**	678	0.26 (0.09–0.71)	**8**.**52E**–**03**
Pathological tumor size									
cm	210	1.00 (0.96–1.04)	0.898						
Pathological FNCLCC grade									
3 vs 1–2	307	1.43 (0.95–2.17)	0.088						
CINSARC risk									
High vs Low	678	2.48 (1.87–3.28)	**2**.**03E**–**10**	678	2.18 (1.63–2.91)	**1**.**24E**–**07**	678	2.18 (1.63–2.91)	**1**.**21E**–**07**
PARP1 classes									
High vs Low	678	1.60 (1.22–2.11)	**6**.**56E**–**04**	678	1.41 (1.06–1.87)	**1**.**86E**–**02**			
PARP1 expression									
Continuous value	678	1.31 (1.09–1.57)	**3**.**45E**–**03**				678	1.24 (1.02–1.51)	**3**.**16E**–**02**

*P*‐values in bold mean significant *P*‐values.

### 3.5 *PARP1* expression and associated biological processes

**Figure 3:**
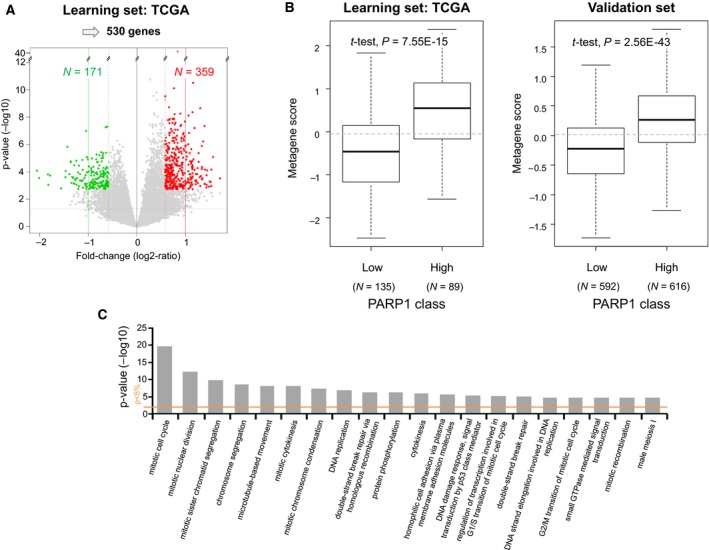
Supervised analysis of gene expression profiles between the ‘PARP1‐high’ and ‘PARP1‐low’ STS classes. (A) Volcano plot showing the 530 genes differentially expressed in the learning set (TCGA). (B) The metagene‐based prediction score is significantly higher (Student’s *t*‐test) in the ‘PARP1‐high’ samples than in the ‘PARP1‐low’ samples in the learning set as expected (left), but also in the independent validation set (right). (C) GO biological processes of the DAVID database associated with the 530‐gene PARP1‐expression signature. The barplot indicates the –log(*P*‐value) (*y*‐axis) of the top 20 biological pathways (*x*‐axis) that are enriched for genes overexpressed in the ‘PARP1‐high’ samples vs the ‘PARP1‐low’ samples. The *P*‐value threshold is indicated by the orange horizontal line.

To further explore the biological alterations associated with the PARP1 expression status, we compared the whole‐genome expression profiles of the ‘PARP1‐high’ (*N* = 89) and ‘PARP1‐low’ (*N* = 135) primary tumor samples in the TCGA data set (Fig. 3A). We identified 530 genes differentially expressed, including 359 genes overexpressed and 171 genes underexpressed in the ‘PARP1‐high’ class (Table S2). The robustness of this gene signature was confirmed in the pool of all other independent sets (1208 primary tumors) by using a metagene‐based prediction score (Fig. 3B): The score was higher in the ‘PARP1‐high’ samples than in the ‘PARP1‐low’ samples (*P* = 2.56E‐43, Student’s *t*‐test). Ontology analysis (Table S3, Fig. 3C) showed strong involvement of genes overexpressed in the ‘PARP1‐high’ samples in cell cycle, chromosome segregation, DNA replication, and DNA repair.

## 4 Discussion

The need for new therapeutic and/or prognostic targets is crucial in STSs. Because of the promising therapeutic value of PARP inhibitors in oncology and the paucity of data in the literature, we analyzed *PARP1* mRNA expression in 1432 previously untreated operated STS samples and 32 relapses. We showed that higher expression was an independent negative prognostic factor for MFS of patients with primary tumors. To our knowledge, this is by far the largest study analyzing *PARP1* expression in STSs.

*PARP1* tumor expression was heterogeneous between samples. The analysis of the 224 TCGA primary tumor samples profiled at both RNA and DNA levels revealed a positive correlation between *PARP1* mRNA expression and DNA copy number; however, gain/amplification was not the sole mechanism of high expression, which was also found in tumors without such alterations. Of note, expression was similar in primary and secondary tumors. No *PARP1* mutation was reported in those 244 primary tumors, nor in the 1215 metastatic samples of the GENIE AACR database (data not shown). The frequency of *PARP1* amplification was also very low in the TCGA primary tumors (0.8%) and the GENIE metastatic samples (0.2%; data not shown).

This wide range of expression values within the 1432 operated primary tumors provided opportunity to search for correlations with clinicopathological features. Our analysis was based on discrete values using the median *PARP1* expression level across the 1432 samples as cut‐off, but similar correlations were found with continuous values. An optimal expression cut‐off able to stratify patients on MFS was measured by means of ROC analysis at 11.62 in the learning set (*N* = 340 samples), very close to our median cut‐off measured at 11.64 (data not shown). It was validated in the validation set (*N* = 338 samples) with significant MFS difference between the ‘PARP1‐high’ vs ‘PARP1‐low’ classes (data not shown). The concordance rate between the two classifications in the whole series was very high, equal to 98.5%. Correlations existed between the two PARP1 classes (‘high’ and ‘low’) and patients’ age, tumor site, pathological type and grade, the CINSARC classification and a chromosomal instability signature, with younger patients in the ‘PARP1‐high’ class, more frequent tumor locations at the extremities, superficial trunk and head and neck, more leiomyosarcomas and other STSs and less liposarcomas and myxofibrosarcomas, more pathological grade 3, more high‐risk CINSARC tumors, and more ‘chromosomically instable’ tumors. Such association with adverse prognostic features was confirmed in univariate analysis with shorter MFS in the ‘PARP1‐high’ class. This negative prognostic value remained significant—suggesting independence—in multivariate analysis. It was notably independent from the pathological type. Interestingly, the prognostic value was observed in liposarcomas and undifferentiated sarcomas, but not in leiomyosarcomas. The fact that one marker has a prognostic value different according to the pathological type of STM is not surprising given the big intertype differences. In liposarcomas, the PARP1‐based classification was associated with the pathological subtypes (well differentiated/dedifferentiated, myxoid, pleomorphic), but its prognostic value persisted in multivariate analysis including these later (data not shown). In leiomyosarcomas, CINSARC was associated with the PARP1‐based classification and with MFS, whereas strikingly PARP1‐based classification had no prognostic value. In undifferentiated sarcomas, none clinicopathological prognostic variable was associated with the PARP1‐based classification that was itself associated with MFS. The analysis of larger series of samples per pathological type is required to better understand such differences. For several decades, efforts have been made to improve the prognostic classification of STSs; different tumor cell‐intrinsic molecular parameters have been proposed, mainly related to cell cycle such as CINSARC (Chibon *et al.*, 2010), as well as parameters related to immune microenvironment, such as *PDL1/CD274* expression (Bertucci *et al.*, 2017). The Fig. 2 suggests the complementary prognostic value of *PARP1* expression (DNA repair) to that of CINSARC and *PDL1* expression. A multivariate analysis of CINSARC, *PDL1* expression, and *PARP1* expression based on the Akaike information criterion retained the CINSARC/*PDL1/PARP1* combination as the best MFS predictive model (*P* = 5.99E‐11; data not shown). Whether the prognostic value of PARP1 expression reflects the metastatic risk and/or the response to eventual adjuvant chemotherapy deserves analysis of a larger and informative series of patients. Indeed, information about delivery or not of adjuvant chemotherapy was available for only 374 out of 678 patients (29 with and 345 without chemotherapy). This is a bias of retrospective analyses. The analysis of MFS in the 345 chemotherapy‐untreated cases showed a trend (*P* = 0.067, log‐rank test) for longer MFS in the ‘PARP1‐low’ class (71% 5‐year MFS, 95% CI: 64–78) than in the ‘PARP1‐high’ class (60% 5‐year MFS, 95% CI: 50–73). No MFS analysis could be done in the 29 patients treated with adjuvant chemotherapy because of the small series size, thus impeding interaction analysis. Considering the multiple functions of PARP1 including cell proliferation and DNA repair, it was not surprising to find high expression associated with shorter MFS, as already reported in other cancers (Goncalves *et al.*, 2011; Li *et al.*, 2016; Mahe *et al.*, 2015; Michels *et al.*, 2015; Murnyak *et al.*, 2017). Regarding STSs, to our knowledge, only two studies have described *PARP1* expression in clinical samples (Kim *et al.*, 2016; Kivlin *et al.*, 2016). All were based on IHC and tissue microarrays and used different antibodies, scoring systems and cut‐offs. In the MDA Cancer Center study (Kivlin *et al.*, 2016), 91 MPNSTs and 24 neurofibromas were analyzed: Overall, MPNST samples had higher levels of *PARP1* expression than neurofibromas; no correlation with clinicopathological features was reported except with survival, which was nonsignificantly higher in MPNSTs with high vs low expression. The authors concluded that ubiquitous expression pattern of *PARP1* supports the use of PARP inhibitors in MPNSTs, but that larger series of samples needed to be analyzed. In the Korean study (Kim *et al.*, 2016), 112 STSs, representing 17 different pathological types, were tested. As found in our study, leiomyosarcomas, undifferentiated sarcomas, and other types were more frequently PARP1‐positive than liposarcomas and myxofibrosarcomas; high expression was associated with higher pathological grade and higher mitotic count; and *PARP1* expression was an independent negative prognostic feature regarding event‐free survival and disease‐specific survival. Of course, the role of such an overexpression in STS initiation or progression, if any, remains to be elucidated. As expected, given the role of PARP1, many genes identified in our supervised analysis as overexpressed in the ‘PARP1‐high’ samples were associated with cell proliferation, which could in part explain such poor prognostic value. However, the *PARP1* expression prognostic value remained independent from the CINSARC signature, possibly reflecting the impact of a reaction against genetic instability and DNA damages. Indeed, several genes involved in DNA repair were overexpressed in the ‘PARP1‐high’ samples, as previously reported in STSs (Kim *et al.*, 2016). Such association of genes overexpressed in the ‘PARP1‐high’ samples with ontologies representing known functions of PARP1 protein provides indication that increased *PARP1* mRNA expression in STS is likely associated with increase in its biological activity and thus its protein expression. The correlation between mRNA and protein expression is also corroborated by the finding of similar clinicopathological correlations of PARP1 expression at the protein level in the Korean series (Kim *et al.*, 2016) and the mRNA level in our present series.

## 5 Conclusion

We showed that *PARP1* mRNA expression is heterogeneous in STS and associated with metastatic relapse independently from the other prognostic features, including the proliferation‐associated CINSARC signature, the most robust prognostic signature reported to date in STSs. The strength of our study lies in the size of the series (the largest series of tumors reported to date regarding analysis of PARP1 expression), its originality (the first one describing *PARP1* mRNA expression in STSs), and the biological and clinical relevance of *PARP1* expression and its independent prognostic value. Limitations include its retrospective multicentric nature and associated biases such as lack of information about the time interval between imaging used during follow‐up, and possible heterogeneity across patients, the heterogeneity with several different STS pathological types, and a limited number of cases in certain types. No overall survival analysis could be done because of the lack of information both quantitative and qualitative. The analysis of larger series, retrospective, then prospective, is warranted to confirm our observation and to assess each pathological type independently. If such prognostic value is confirmed, *PARP1* expression might refine the prediction of metastatic relapse and improve our ability to tailor adjuvant chemotherapy. Given this unfavorable prognostic value, STS patients with high level of *PARP1* expression would warrant a more aggressive treatment plan, which might include PARP1 inhibitors possibly associated with trabectedin given the predictive value of high *PARP1* expression (Grignani *et al.*, 2018; Pignochino *et al.*, 2017) or with other DNA‐damaging agents. We found similar expression level in secondary vs primary tumors. Even if analysis of larger series of metastatic samples is warranted, our results support the ongoing development of PARP inhibitors in STSs. In the future, it will be important not only to test whether *PARP1* mRNA expression can predict the clinical response to PARP1 inhibitors or DNA‐damaging agents, but also to validate our findings at the protein level using IHC that remains more convenient for use in clinical routine.

## Conflict of interest

The authors declare no conflict of interest.

## Author contributions

FB was involved in the conception and design of the work, acquisition, analysis and interpretation of data, and draft of the manuscript. PF was involved in the acquisition, analysis, and interpretation of all data. AM, DP, CC, ALC, JYB, OM, and DB were involved in the acquisition, analysis, and interpretation of data. DB was also involved in the draft of the manuscript. All authors read critically and approved the final manuscript.

## Supporting information


**Fig. S1**. MFS in patients with different STS pathological types according to *PARP1* expression. (A) Kaplan‐Meier MFS curves in 256 patients with liposarcoma, according to the PARP1‐based classification (‘PARP1‐low’ and ‘PARP1‐high’ classes). (B) Similar to A, but in 202 patients with undifferentiated sarcoma. (C) Similar to A, but in 149 patients with leiomyosarcoma.

**Fig. S2**. MFS in patients with STS according to *PARP1* expression, CINSARC signature, and *PDL1* expression. Kaplan‐Meier MFS curves in 470 patients with STS, informative for the three variables: *PARP1* expression (high and low), CINSARC signature (high‐risk and low‐risk), and *PDL1* expression (high and low). The PDL1 legend and the colors in the table to the right of the figure define the eight patients groups.

**Table S1**. List of soft tissue sarcoma data sets included.

**Table S2**. List of 530 genes differentially expressed between the ‘PARP1‐high’ and ‘PARP1‐low’ sample classes.

**Table S3**. Ontology analysis of the 530 genes differentially expressed between the ‘PARP1‐high’ and ‘PARP1‐low’ sample classes.

## Data Availability

All data are available in the National Center for Biotechnology Information (NCBI)/GenBank GEO and ArrayExpress databases and authors’ web sites as indicated in Table S1.

## References

[mol212522-bib-0001] Ame JC , Spenlehauer C and de Murcia G (2004) The PARP superfamily. BioEssays 26, 882–893. 15273990 10.1002/bies.20085

[mol212522-bib-0002] Baird K , Davis S , Antonescu CR , Harper UL , Walker RL , Chen Y , Glatfelter AA , Duray PH and Meltzer PS (2005) Gene expression profiling of human sarcomas: insights into sarcoma biology. Cancer Res 65, 9226–9235. 16230383 10.1158/0008-5472.CAN-05-1699

[mol212522-bib-0003] Barretina J , Taylor BS , Banerji S , Ramos AH , Lagos‐Quintana M , Decarolis PL , Shah K , Socci ND , Weir BA , Ho A *et al* (2010) Subtype‐specific genomic alterations define new targets for soft‐tissue sarcoma therapy. Nat Genet 42, 715–721. 20601955 10.1038/ng.619 PMC2911503

[mol212522-bib-0004] Beck AH , Lee CH , Witten DM , Gleason BC , Edris B , Espinosa I , Zhu S , Li R , Montgomery KD , Marinelli RJ *et al* (2010) Discovery of molecular subtypes in leiomyosarcoma through integrative molecular profiling. Oncogene 29, 845–854. 19901961 10.1038/onc.2009.381 PMC2820592

[mol212522-bib-0005] Bertucci F , Finetti P , Perrot D , Leroux A , Collin F , Le Cesne A , Coindre JM , Blay JY , Birnbaum D and Mamessier E (2017) PDL1 expression is a poor‐prognosis factor in soft‐tissue sarcomas. Oncoimmunology 6, e1278100. 28405501 10.1080/2162402X.2016.1278100 PMC5384364

[mol212522-bib-0006] Bertucci F , Finetti P , Viens P and Birnbaum D (2014) EndoPredict predicts for the response to neoadjuvant chemotherapy in ER‐positive, HER2‐negative breast cancer. Cancer Lett 355, 70–75. 25218596 10.1016/j.canlet.2014.09.014

[mol212522-bib-0007] Bryant HE , Schultz N , Thomas HD , Parker KM , Flower D , Lopez E , Kyle S , Meuth M , Curtin NJ and Helleday T (2005) Specific killing of BRCA2‐deficient tumours with inhibitors of poly(ADP‐ribose) polymerase. Nature 434, 913–917. 15829966 10.1038/nature03443

[mol212522-bib-0008] Cancer Genome Atlas Research Network (2017) Comprehensive and integrated genomic characterization of adult soft tissue sarcomas. Cell 171(950–965), e928. 10.1016/j.cell.2017.10.014 PMC5693358 29100075

[mol212522-bib-0009] Carter SL , Eklund AC , Kohane IS , Harris LN and Szallasi Z (2006) A signature of chromosomal instability inferred from gene expression profiles predicts clinical outcome in multiple human cancers. Nat Genet 38, 1043–1048. 16921376 10.1038/ng1861

[mol212522-bib-0010] Casali PG , Abecassis N , Bauer S , Biagini R , Bielack S , Bonvalot S , Boukovinas I , Bovee J , Brodowicz T and Broto JM *et al* (2018) Soft tissue and visceral sarcomas: ESMO‐EURACAN Clinical Practice Guidelines for diagnosis, treatment and follow‐up. Ann Oncol 29, iv51–iv67. 29846498 10.1093/annonc/mdy096

[mol212522-bib-0011] Chibon F , Lagarde P , Salas S , Perot G , Brouste V , Tirode F , Lucchesi C , de Reynies A , Kauffmann A , Bui B *et al* (2010) Validated prediction of clinical outcome in sarcomas and multiple types of cancer on the basis of a gene expression signature related to genome complexity. Nat Med 16, 781–787. 20581836 10.1038/nm.2174

[mol212522-bib-0012] Chudasama P , Mughal SS , Sanders MA , Hubschmann D , Chung I , Deeg KI , Wong SH , Rabe S , Hlevnjak M , Zapatka M *et al* (2018) Integrative genomic and transcriptomic analysis of leiomyosarcoma. Nat Commun 9, 144. 29321523 10.1038/s41467-017-02602-0 PMC5762758

[mol212522-bib-0013] Detwiller KY , Fernando NT , Segal NH , Ryeom SW , D'Amore PA and Yoon SS (2005) Analysis of hypoxia‐related gene expression in sarcomas and effect of hypoxia on RNA interference of vascular endothelial cell growth factor A. Cancer Res 65, 5881–5889. 15994966 10.1158/0008-5472.CAN-04-4078

[mol212522-bib-0014] Farmer H , McCabe N , Lord CJ , Tutt AN , Johnson DA , Richardson TB , Santarosa M , Dillon KJ , Hickson I , Knights C *et al* (2005) Targeting the DNA repair defect in BRCA mutant cells as a therapeutic strategy. Nature 434, 917–921. 15829967 10.1038/nature03445

[mol212522-bib-0015] Gibault L , Perot G , Chibon F , Bonnin S , Lagarde P , Terrier P , Coindre JM and Aurias A (2011) New insights in sarcoma oncogenesis: a comprehensive analysis of a large series of 160 soft tissue sarcomas with complex genomics. J Pathol 223, 64–71. 21125665 10.1002/path.2787

[mol212522-bib-0016] Gobble RM , Qin LX , Brill ER , Angeles CV , Ugras S , O'Connor RB , Moraco NH , Decarolis PL , Antonescu C and Singer S (2011) Expression profiling of liposarcoma yields a multigene predictor of patient outcome and identifies genes that contribute to liposarcomagenesis. Cancer Res 71, 2697–2705. 21335544 10.1158/0008-5472.CAN-10-3588 PMC3070774

[mol212522-bib-0017] Goncalves A , Finetti P , Sabatier R , Gilabert M , Adelaide J , Borg JP , Chaffanet M , Viens P , Birnbaum D and Bertucci F (2011) Poly(ADP‐ribose) polymerase‐1 mRNA expression in human breast cancer: a meta‐analysis. Breast Cancer Res Treat 127, 273–281. 21069454 10.1007/s10549-010-1199-y

[mol212522-bib-0018] Grignani G , D'Ambrosio L , Pignochino Y , Palmerini E , Zucchetti M , Boccone P , Aliberti S , Stacchiotti S , Bertulli R , Piana R *et al* (2018) Trabectedin and olaparib in patients with advanced and non‐resectable bone and soft‐tissue sarcomas (TOMAS): an open‐label, phase 1b study from the Italian Sarcoma Group. Lancet Oncol 19, 1360–1371. 30217671 10.1016/S1470-2045(18)30438-8

[mol212522-bib-0019] Hajdu M , Singer S , Maki RG , Schwartz GK , Keohan ML and Antonescu CR (2010) IGF2 over‐expression in solitary fibrous tumours is independent of anatomical location and is related to loss of imprinting. J Pathol 221, 300–307. 20527023 10.1002/path.2715 PMC3264680

[mol212522-bib-0020] Henderson SR , Guiliano D , Presneau N , McLean S , Frow R , Vujovic S , Anderson J , Sebire N , Whelan J , Athanasou N *et al* (2005) A molecular map of mesenchymal tumors. Genome Biol 6, R76. 16168083 10.1186/gb-2005-6-9-r76 PMC1242211

[mol212522-bib-0021] Hochberg Y and Benjamini Y (1990) More powerful procedures for multiple significance testing. Stat Med 9, 811–818. 2218183 10.1002/sim.4780090710

[mol212522-bib-0022] Irizarry RA , Hobbs B , Collin F , Beazer‐Barclay YD , Antonellis KJ , Scherf U and Speed TP (2003) Exploration, normalization, and summaries of high density oligonucleotide array probe level data. Biostatistics 4, 249–264. 12925520 10.1093/biostatistics/4.2.249

[mol212522-bib-0023] Kim KM , Moon YJ , Park SH , Park HJ , Wang SI , Park HS , Lee H , Kwon KS , Moon WS , Lee DG *et al* (2016) Individual and combined expression of DNA damage response molecules PARP1, gammaH2AX, BRCA1, and BRCA2 predict shorter survival of soft tissue sarcoma patients. PLoS ONE 11, e0163193. 27643881 10.1371/journal.pone.0163193 PMC5028069

[mol212522-bib-0024] Kivlin CM , Watson KL , Al Sannaa GA , Belousov R , Ingram DR , Huang KL , May CD , Bolshakov S , Landers SM , Kalam AA *et al* (2016) Poly (ADP) ribose polymerase inhibition: a potential treatment of malignant peripheral nerve sheath tumor. Cancer Biol Ther 17, 129–138. 26650448 10.1080/15384047.2015.1108486 PMC4847988

[mol212522-bib-0025] Laroche A , Chaire V , Le Loarer F , Algeo MP , Rey C , Tran K , Lucchesi C and Italiano A (2017) Activity of trabectedin and the PARP inhibitor rucaparib in soft‐tissue sarcomas. J Hematol Oncol 10, 84. 28399901 10.1186/s13045-017-0451-x PMC5387279

[mol212522-bib-0026] Li Z , Lv T , Liu Y , Huang X , Qiu Z and Li J (2016) PARP1 is a novel independent prognostic factor for the poor prognosis of chordoma. Cancer Biomark 16, 633–639. 27002766 10.3233/CBM-160605 PMC13016521

[mol212522-bib-0027] Lim JSJ and Tan DSP (2017) Understanding resistance mechanisms and expanding the therapeutic utility of PARP inhibitors. Cancers 9, pii: E109. 10.3390/cancers9080109 PMC5575612 28829366

[mol212522-bib-0028] Mahe E , Akhter A , Le A , Street L , Pournaziri P , Kosari F , Shabani‐Rad MT , Stewart D and Mansoor A (2015) PARP1 expression in mantle cell lymphoma: the utility of PARP1 immunohistochemistry and its relationship with markers of DNA damage. Hematol Oncol 33, 159–165. 10.1002/hon.2160 25143154

[mol212522-bib-0029] McShane LM , Altman DG , Sauerbrei W , Taube SE , Gion M , Clark GM and Statistics Subcommittee of the NCIEWGoCD . (2005) Reporting recommendations for tumour MARKer prognostic studies (REMARK). Br J Cancer 93, 387–391. 16106245 10.1038/sj.bjc.6602678 PMC2361579

[mol212522-bib-0030] Michels J , Adam J , Goubar A , Obrist F , Damotte D , Robin A , Alifano M , Vitale I , Olaussen KA , Girard P *et al* (2015) Negative prognostic value of high levels of intracellular poly(ADP‐ribose) in non‐small cell lung cancer. Ann Oncol 26, 2470–2477. 26387143 10.1093/annonc/mdv393

[mol212522-bib-0031] Murnyak B , Kouhsari MC , Hershkovitch R , Kalman B , Marko‐Varga G , Klekner A and Hortobagyi T (2017) PARP1 expression and its correlation with survival is tumour molecular subtype dependent in glioblastoma. Oncotarget 8, 46348–46362. 28654422 10.18632/oncotarget.18013 PMC5542272

[mol212522-bib-0032] Nakayama R , Nemoto T , Takahashi H , Ohta T , Kawai A , Seki K , Yoshida T , Toyama Y , Ichikawa H and Hasegawa T (2007) Gene expression analysis of soft tissue sarcomas: characterization and reclassification of malignant fibrous histiocytoma. Mod Pathol 20, 749–759. 17464315 10.1038/modpathol.3800794

[mol212522-bib-0033] Nielsen TO , West RB , Linn SC , Alter O , Knowling MA , O'Connell JX , Zhu S , Fero M , Sherlock G , Pollack JR *et al* (2002) Molecular characterisation of soft tissue tumours: a gene expression study. Lancet 359, 1301–1307. 11965276 10.1016/S0140-6736(02)08270-3

[mol212522-bib-0034] Pignochino Y , Capozzi F , D'Ambrosio L , Dell'Aglio C , Basirico M , Canta M , Lorenzato A , Vignolo Lutati F , Aliberti S , Palesandro E *et al* (2017) PARP1 expression drives the synergistic antitumor activity of trabectedin and PARP1 inhibitors in sarcoma preclinical models. Mol Cancer 16, 86. 28454547 10.1186/s12943-017-0652-5 PMC5410089

[mol212522-bib-0035] Renner M , Wolf T , Meyer H , Hartmann W , Penzel R , Ulrich A , Lehner B , Hovestadt V , Czwan E , Egerer G *et al* (2013) Integrative DNA methylation and gene expression analysis in high‐grade soft tissue sarcomas. Genome Biol 14, r137. 24345474 10.1186/gb-2013-14-12-r137 PMC4054884

[mol212522-bib-0036] Skubitz KM , Francis P , Skubitz AP , Luo X and Nilbert M (2012) Gene expression identifies heterogeneity of metastatic propensity in high‐grade soft tissue sarcomas. Cancer 118, 4235–4243. 22252777 10.1002/cncr.26733

[mol212522-bib-0037] Stewart E , Goshorn R , Bradley C , Griffiths LM , Benavente C , Twarog NR , Miller GM , Caufield W , Freeman BB 3rd , Bahrami A *et al* (2014) Targeting the DNA repair pathway in Ewing sarcoma. Cell Rep 9, 829–841. 25437539 10.1016/j.celrep.2014.09.028 PMC4386669

[mol212522-bib-0038] Vormoor B and Curtin NJ (2014) Poly(ADP‐ribose) polymerase inhibitors in Ewing sarcoma. Curr Opin Oncol 26, 428–433. 24840521 10.1097/CCO.0000000000000091 PMC4059819

[mol212522-bib-0039] West RB , Nuyten DS , Subramanian S , Nielsen TO , Corless CL , Rubin BP , Montgomery K , Zhu S , Patel R , Hernandez‐Boussard T *et al* (2005) Determination of stromal signatures in breast carcinoma. PLoS Biol 3, e187. 15869330 10.1371/journal.pbio.0030187 PMC1088973

[mol212522-bib-0040] Ylipaa A , Hunt KK , Yang J , Lazar AJ , Torres KE , Lev DC , Nykter M , Pollock RE , Trent J and Zhang W (2011) Integrative genomic characterization and a genomic staging system for gastrointestinal stromal tumors. Cancer 117, 380–389. 20818650 10.1002/cncr.25594 PMC3008331

