# Supplementary material for: PARP1 expression in soft tissue sarcomas is a poor‐prognosis factor and a new potential therapeutic target
Source: Mol Oncol. 2019 Jun 7;13(7):1577–88. doi: 10.1002/1878-0261.12522 (PMC6599836; doi:10.1002/1878-0261.12522)
Supplement: Supplementary file 1 — Fig. S1. MFS in patients with different STS pathological types according to PARP1 expression. (A) Kaplan‐Meier MFS curves in 256 patients with liposarcoma, according to the PARP1‐based classification (‘PARP1‐low’ and ‘PARP1‐high’ classes). (B) Similar to A, but in 202 patients with undifferentiated sarcoma. (C) Similar to A, but in 149 patients with leiomyosarcoma. [file MOL2-13-1577-s001.pptx]

## Slide 1
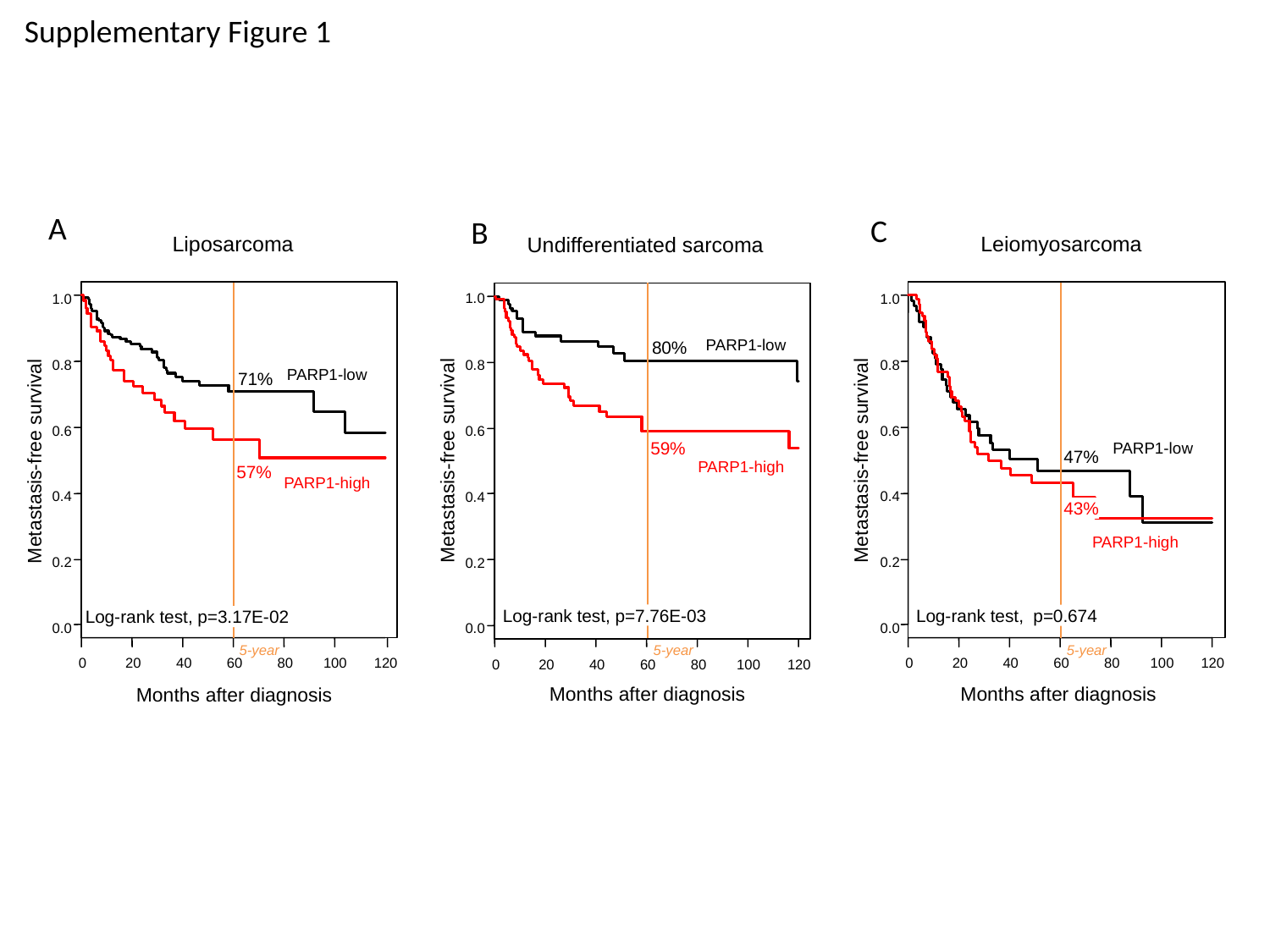

Supplementary Figure 1
A
Liposarcoma
1.0
0.8
PARP1-low
71%
0.6
Metastasis-free survival
57%
PARP1-high
0.4
0.2
Log-rank test, p=3.17E-02
0.0
5-year
0
20
40
60
80
100
120
Months after diagnosis
C
Leiomyosarcoma
1.0
0.8
0.6
PARP1-low
47%
Metastasis-free survival
0.4
43%
PARP1-high
0.2
Log-rank test, p=0.674
0.0
5-year
0
20
40
60
80
100
120
Months after diagnosis
B
Undifferentiated sarcoma
1.0
PARP1-low
80%
0.8
0.6
59%
Metastasis-free survival
PARP1-high
0.4
0.2
Log-rank test, p=7.76E-03
0.0
5-year
0
20
40
60
80
100
120
Months after diagnosis
