# Supplementary material for: PARP1 expression in soft tissue sarcomas is a poor‐prognosis factor and a new potential therapeutic target
Source: Mol Oncol. 2019 Jun 7;13(7):1577–88. doi: 10.1002/1878-0261.12522 (PMC6599836; doi:10.1002/1878-0261.12522)
Supplement: Supplementary file 2 — Fig. S2. MFS in patients with STS according to PARP1 expression, CINSARC signature, and PDL1 expression. Kaplan‐Meier MFS curves in 470 patients with STS, informative for the three variables: PARP1 expression (high and low), CINSARC signature (high‐risk and low‐risk), and PDL1 expression (high and low). The PDL1 legend and the colors in the table to the right of the figure define the eight patients groups. [file MOL2-13-1577-s002.pptx]

## Slide 1
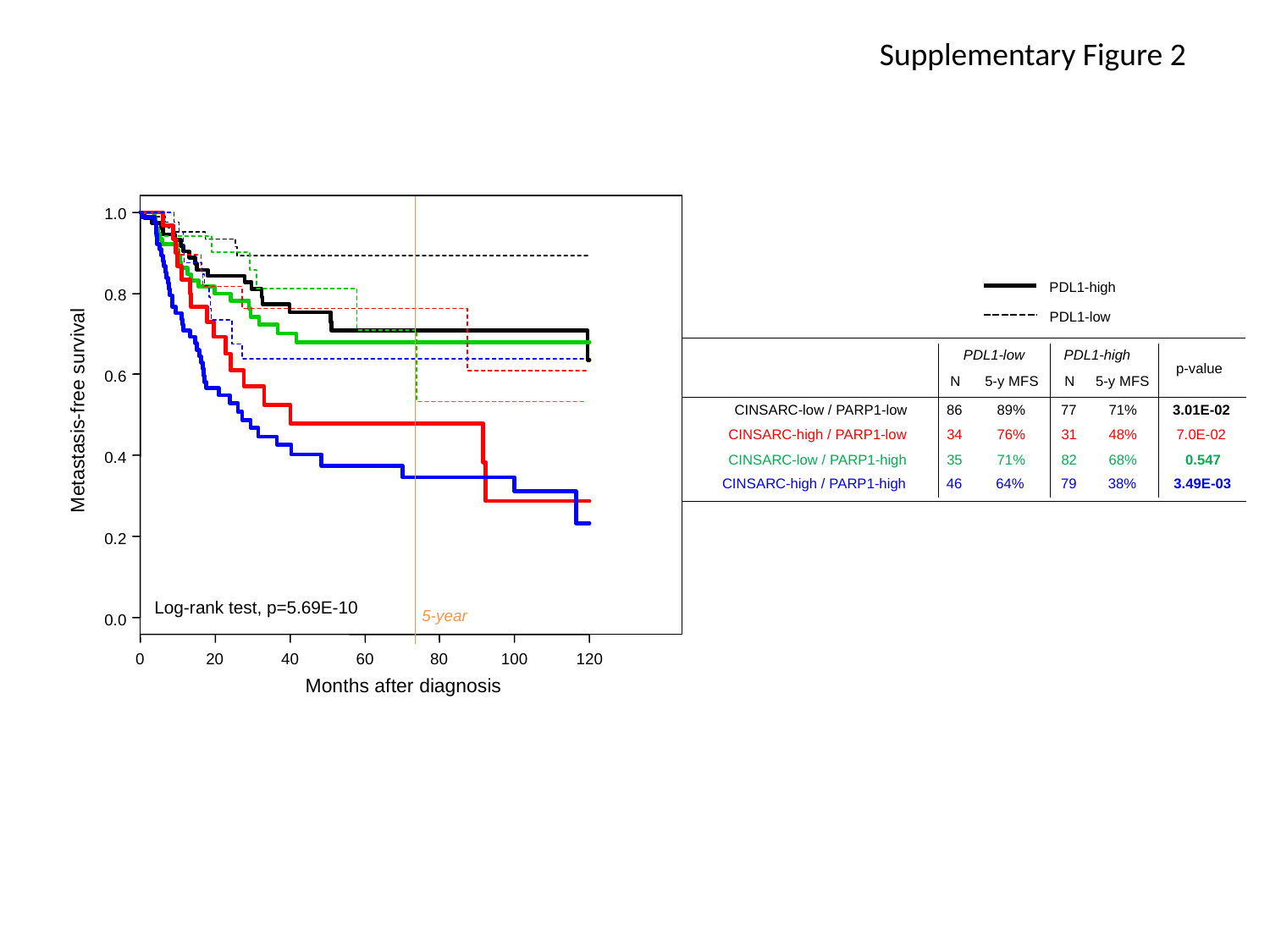

Supplementary Figure 2
1.0
0.8
0.6
Metastasis-free survival
0.4
0.2
Log-rank test, p=5.69E-10
0.0
0
20
40
60
80
100
120
Months after diagnosis
PDL1-high
PDL1-low
PDL1-low
PDL1-high
p-value
N
5-y MFS
N
5-y MFS
CINSARC-low / PARP1-low
86
89%
77
71%
3.01E-02
CINSARC-high / PARP1-low
34
76%
31
48%
7.0E-02
CINSARC-low / PARP1-high
35
71%
82
68%
0.547
CINSARC-high / PARP1-high
46
64%
79
38%
3.49E-03
5-year
